# RAxML and FastTree: Comparing Two Methods for Large-Scale Maximum Likelihood Phylogeny Estimation

**DOI:** 10.1371/journal.pone.0027731

**Published:** 2011-11-21

**Authors:** Kevin Liu, C. Randal Linder, Tandy Warnow

**Affiliations:** 1 Department of Computer Science, University of Texas at Austin, Austin, Texas, United States of America; 2 Section of Integrative Biology, School of Biological Sciences, University of Texas at Austin, Austin, Texas, United States of America; Pennsylvania State University, United States of America

## Abstract

Statistical methods for phylogeny estimation, especially maximum likelihood (ML), offer high accuracy with excellent theoretical properties. However, RAxML, the current leading method for large-scale ML estimation, can require weeks or longer when used on datasets with thousands of molecular sequences. Faster methods for ML estimation, among them FastTree, have also been developed, but their relative performance to RAxML is not yet fully understood. In this study, we explore the performance with respect to ML score, running time, and topological accuracy, of FastTree and RAxML on thousands of alignments (based on both simulated and biological nucleotide datasets) with up to 27,634 sequences. We find that when RAxML and FastTree are constrained to the same running time, FastTree produces topologically much more accurate trees in almost all cases. We also find that when RAxML is allowed to run to completion, it provides an advantage over FastTree in terms of the ML score, but does not produce substantially more accurate tree topologies. Interestingly, the relative accuracy of trees computed using FastTree and RAxML depends in part on the accuracy of the sequence alignment and dataset size, so that FastTree can be more accurate than RAxML on large datasets with relatively inaccurate alignments. Finally, the running times of RAxML and FastTree are dramatically different, so that when run to completion, RAxML can take several orders of magnitude longer than FastTree to complete. Thus, our study shows that very large phylogenies can be estimated very quickly using FastTree, with little (and in some cases no) degradation in tree accuracy, as compared to RAxML.

## Introduction

Phylogeny estimation is an important part of much biological research. Methods (either Bayesian or maximum likelihood) based upon stochastic models of sequence evolution have many desirable statistical properties, but are also computationally the most challenging. Bayesian MCMC methods (e.g., MrBayes [1]) offer an advantage over maximum likelihood in that they provide a distribution of trees rather than a single point estimate; however, because the time needed for the MCMC analysis to converge can be very large, these methods are generally not used on datasets with more than a few hundred sequences. Large-scale statistical phylogeny estimation, with many hundreds or several thousand sequences, therefore, is performed using maximum likelihood (ML). Of the many ML methods, RAxML [2], [3] is the main method for large-scale ML estimation because it produces the best ML scores and does so faster than other ML methods that have comparable accuracy with respect to ML scores. Other widely-used ML methods include GARLI [4], Phyml [5], and PAUP* [6], but these methods have generally not been used on very large datasets.

Although continuous enhancements are being added to RAxML, its computational requirements can still be prohibitive for alignments with more than a few thousand sequences and sites (e.g., a RAxML analysis of several alignments of a 16S dataset with almost 28,000 sequences required approximately a month of CPU time [7], and a RAxML bootstrap analysis of that dataset with 444 replicates required 5.6 *years* of CPU time [8], [9]). Faster methods for finding trees with good ML scores also exist, one of which is FastTree [10], [11]. Earlier studies comparing FastTree to RAxML on true alignments showed that RAxML was much more computationally intensive than FastTree, and that RAxML produced better ML scores and topologically more accurate trees than FastTree [11]. However, the relative performance on estimated alignments was not evaluated, nor was any comparison made between FastTree and RAxML when restricted to the same amount of time.

In this paper, we compare RAxML and FastTree on nucleotide datasets, when alignments must be estimated. We explore performance with respect to running time, ML score, and topological accuracy, using both biological and simulated datasets and estimating alignments using several different methods. Because running time is a crucial issue for large datasets, we include a variant of RAxML (which we cal “RAxML-Limited”), in which we constrain RAxML's running time so that it is not substantially longer than FastTree's.

Our study shows that in many cases, phylogenetic analyses of very large nucleotide alignments can be performed using FastTree without a substantial difference in tree accuracy, and in a small fraction of the time needed by RAxML. Thus, FastTree represents an important contribution achievement in the state of the art for ML tree estimation on nucleotide sequence alignments.

## Results

### Simulated Data

We compared RAxML, RAxML-Limited, and FastTree on 1800 1000-taxon alignments, previously studied in [8], [9] and available online at www.cs.utexas.edu/users/tandy/science-paper.html. These alignment were estimated on simulated datasets (Table S1) produced using ROSE [12] on 15 model trees (with 20 replicates produced per model tree) under different GTR+Gamma+Indel models, with a range of rates of evolution, distributions on gap lengths (short, medium, and long, indicated by S, M, and L in the model names), and relative rates of substitutions to indels. For each dataset, we produced six alignments: the true alignment (known to us because of the simulation process), and using MAFFT, SATé, ClustalW, QuickTree and PartTree, each in their default settings, to estimate alignments. For each of the 1800 resulting alignments, we used RAxML, FastTree, and RAxML-Limited to estimate ML trees. We compared each estimated tree to the true tree (known to us due to the simulation process), with zero-event edges contracted (these are called “potentially inferable model trees”, or PIMTs). We measured topological accuracy using the missing branch rate, which is the proportion of branches (as defined by the bipartitions they induce on the taxon set) present in the PIMT but missing in the estimated tree. When the PIMT is fully resolved, then the missing branch rate is identical to the bipartition distance (also known as the Robinson-Foulds rate), the standard metric used in phylogenetic studies. However, when the PIMT tree is not binary, the missing branch rate is preferable. The missing branch and Robinson-Foulds rates treat all missing branches equally, and hence does not reveal where within the PIMT the missing branches are located.

**Figure 1:**
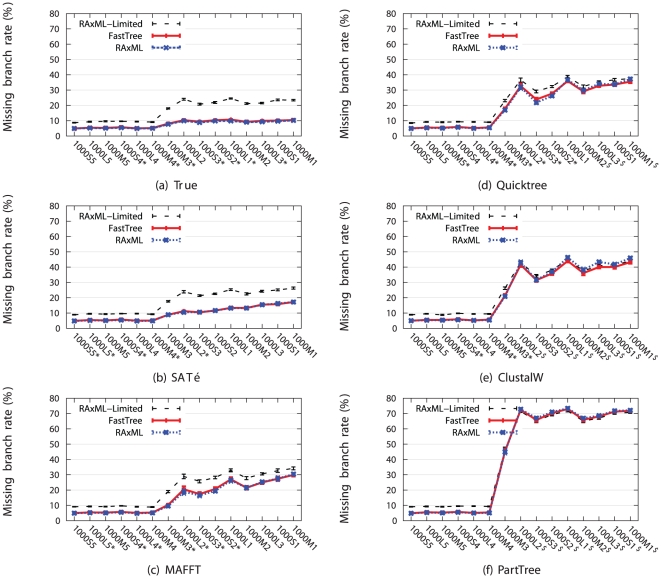
Missing branch rates of ML methods on the simulated 1000-taxon datasets. The 1000-taxon model conditions are arranged along each x-axis from left to right in order of increasing difficulty. Standard error bars are shown. 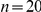 for each average value. Statistical tests were performed using one-tailed pairwise t-tests with Benjamini-Hochberg [13] correction for multiple tests. Model conditions marked with an asterisk (“*”) indicate that RAxML's missing branch rate was a statistically significant improvement over FastTree's missing branch rate. Model conditions marked with a dollar sign (“$”) indicate that FastTree's missing branch rate was a statistically significant improvement over RAxML's missing branch rate. 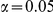 and 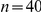 for each statistical test.

Throughout these experiments we observed the following. First, for almost all model conditions and alignment methods, RAxML-Limited produces the least accurate ML scores and tree topologies of all three methods, with results that are generally statistically significant (Figure 1 and Table S2 and S3 for tree error, and Figure S1 and Table S4 and S5 for ML score, Benjamini-Hochberg-corrected [13] one-tailed pairwise t-tests, 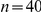 and 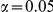 for each statistical test).

Figure 1 presents a comparison with respect to topological accuracy (the more accurate alignments are on the left, and less accurate on the right). Note that RAxML produced more accurate trees than RAxML-Limited for all easy model conditions (1000S5, 1000L5, 1000M5, 1000S4, and 1000L4), on which, irrespective of the alignment, RAxML produced trees with accuracy very close to that of RAxML on the true alignment. On the harder model conditions, RAxML gave a substantial improvement over RAxML-Limited for the more accurate alignments, and a smaller but still distinct improvement on the less accurate alignments.

A comparison between RAxML and FastTree (Fig. 1) with respect to tree topology accuracy shows that RAxML and FastTree had very close accuracy on the easy model conditions for all alignments (differing by at most 0.3%). On the harder models, RAxML produced more accurate trees than FastTree for highly accurate alignments (true, SATé, and MAFFT alignments), and FastTree was able to produce more accurate trees on the less accurate alignments. Thus, the relative accuracy of RAxML and FastTree depended upon the accuracy of the alignment, with RAxML performing better on the more accurate alignments and FastTree giving better results on the less accurate alignments.

However, even on the harder models, the differences were small, and not all models show statistically significant differences. On the more accurate alignments (i.e., the true, SATé, and MAFFT alignments), all the model conditions showing statistically significant differences between RAxML and FastTree favored RAxML. The average improvement on these model conditions was 0.5% on the true alignment, 0.5% on the SATé alignment, and 1.1% on the MAFFT alignment. Thus, although there were statistically significant differences, their magnitudes were small.

On the less accurate alignments, we see some interesting differences. On the ClustalW alignments, nine of the ten harder model conditions showed statistically significant differences between RAxML and FastTree: two showed RAxML having an advantage over FastTree (but with the average improvement only 0.4%), and seven showed FastTree having an advantage over RAxML (average improvement 2.1%). On the Quicktree alignments, eight of the ten model conditions showed statistically significant differences, with three in favor of FastTree (average improvement 1.2%) and five in favor of RAxML (average improvement 1.1%). Finally, on the PartTree alignments, eight of the ten harder model conditions showed statistically significant improvements, all in favor of FastTree (average improvement 0.6%).

Thus, with respect to tree topology accuracy, the relative performance of RAxML and FastTree depended upon both the model parameters and alignment accuracy, RAxML tending to have an advantage on alignments that were highly accurate (easy model conditions or very good alignments on harder model conditions), and FastTree tending to have an advantage otherwise. Furthermore, although many of the differences were statistically significant (see Tables S3 and S6), the differences in tree accuracy were small on average, and differences on individual model conditions were at most 3.4%.

**Figure 2:**
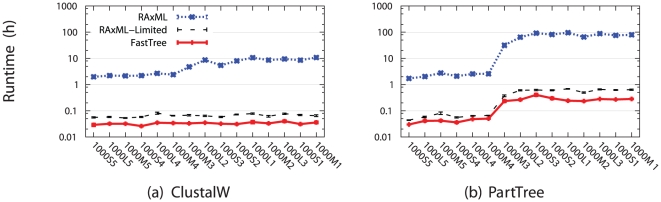
Runtimes (h) of ML methods on the ClustalW and PartTree alignments of the simulated 1000-taxon datasets. Runtimes of ML methods on other alignments are similar to runtimes on the ClustalW alignment (data not shown). The 1000-taxon model conditions are arranged along each x-axis from left to right in order of increasing difficulty. Standard error bars are shown. 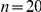 for each reported value.

A comparison of running times shows dramatic differences among these three methods (Fig. 2). Note that both FastTree and RAxML-Limited finished in at most ten minutes on all the datasets we studied, while RAxML's running time was much larger, ranging from about 2 hours to almost 100 hours. Furthermore, RAxML's running time was impacted by the alignment choice, so that RAxML took much longer on the PartTree alignments for the difficult model conditions. The increase in running time for the PartTree alignments on these hard model conditions suggests that RAxML's running time may be due to poor phylogenetic signal in the PartTree alignments, which were the least accurate in our collection for the simulated data.

### Biological datasets

We studied performance on ten ribosomal RNA datasets with 117 to 27,643 sequences from CRW (the Comparative Ribosomal Website produced by Robin Gutell [14]), which have highly reliable curated alignments based upon secondary structure. These datasets had been previously used as benchmarks for alignment and phylogeny estimation [7], and so have curated alignments and reference trees (based upon RAxML bootstrapping analyses with only the highly supported branches included) available. See Table S7 for empirical statistics for these datasets and reference trees.

For each of the datasets, we computed several alignments: the Quicktree and PartTree alignments only on the three largest datasets, and MAFFT, ClustalW, and SATé on the smaller biological datasets. We ran RAxML, RAxML-Limited, and FastTree on these alignments, and compared the resultant trees to the reference tree for each dataset.

With respect to ML score optimization, RAxML produced the best ML scores for all the alignment/dataset combinations (Tables 1 and 2). However, RAxML-Limited produced better ML scores than FastTree on the alignments with at least 1000 sequences, and FastTree produced better ML scores on almost all of the alignments with fewer than 1000 sequences.

**Table 1:** ML scores (as log likelihoods) of the solutions obtained by the ML methods on the three largest biological datasets.

Alignment	ML Method	16S.B.ALL	16S.T	16S.3
TrueAln	RAxML	**−1,589,345**	**−1,727,411**	**−1,376,372**
	FastTree	**−1,607,277**	**−1,731,963**	**−1,380,308**
	RAxML-Limited	**−1,601,407**	**−1,733,148**	**−1,380,946**
SATé	RAxML	**n.d.**	**−1,650,778**	**−1,316,340**
	FastTree	**n.d.**	**−1,654,334**	**−1,319,623**
	RAxML-Limited	**n.d.**	**−1,655,854**	**−1,317,604**
MAFFT	RAxML	**n.d.**	**−1,645,509**	**n.d.**
	FastTree	**n.d.**	**−1,649,615**	**n.d.**
	RAxML-Limited	**n.d.**	**−1,647,176**	**n.d.**
PartTree	RAxML	**−1,973,592**	**−1,760,582**	**−1,346,922**
	FastTree	**−2,032,424**	**−1,766,371**	**−1,350,858**
	RAxML-Limited	**−2,013,115**	**−1,762,611**	**−1,351,822**
ClustalW	RAxML	**n.d.**	**−1,716,012**	**−1,365,614**
	FastTree	**n.d.**	**−1,719,138**	**−1,369,027**
	RAxML-Limited	**n.d.**	**−1,720,420**	**−1,368,776**
Quicktree	RAxML	**−1,878,299**	**−3,439,265**	**−2,451,782**
	FastTree	**−1,903,840**	**−3,449,574**	**−2,459,658**
	RAxML-Limited	**−1,891,570**	**−3,448,084**	**−2,460,095**

Some alignments were missing **due to high memory requirements of an alignment method on datasets of this size**, preventing ML methods from running; these entries are marked “n.d.”. *n* = 1 for each reported value.

**Table 2:** ML scores obtained by the ML methods on the six smallest biological datasets.

Alignment	ML Method	16S.M.aa_ag	16S.M	23S.M	23S.M.aa_ag	23S.E.aa_ag	23S.E
Reference	RAxML	**−279,440**	**−288,263**	**−241,190**	**−226,619**	**−194,711**	**−190,752**
	FastTree	**−279,848**	**−288,626**	**−241,419**	**−226,796**	**−194,856**	**−191,304**
	RAxML-Limited	**−280,385**	**−289,222**	**−241,759**	**−226,921**	**−195,182**	**−191,029**
SATé	RAxML	**−262,698**	**−265,706**	**−229,241**	**−215,823**	**−178,413**	**−179,482**
	FastTree	**−263,142**	**−265,951**	**−229,322**	**−215,864**	**−178,634**	**−179,712**
	RAxML-Limited	**−263,631**	**−266,457**	**−229,670**	**−216,059**	**−178,597**	**−179,622**
MAFFT	RAxML	**−264,947**	**−266,519**	**−233,776**	**−219,249**	**−182,246**	**−181,039**
	FastTree	**−265,463**	**−266,798**	**−233,867**	**−219,310**	**−182,446**	**−181,251**
	RAxML-Limited	**−265,663**	**−267,217**	**−234,113**	**−219,620**	**−182,466**	**−181,404**
PartTree	RAxML	**−270,834**	**−262,794**	**−232,272**	**−216,530**	**−174,603**	**−176,671**
	FastTree	**−271,496**	**−263,032**	**−232,517**	**−216,628**	**−174,739**	**−176,809**
	RAxML-Limited	**−271,642**	**−263,569**	**−232,691**	**−216,881**	**−174,817**	**−177,006**
ClustalW	RAxML	**−279,144**	**−276,110**	**−233,343**	**−220,541**	**−187,819**	**−197,246**
	FastTree	**−279,994**	**−276,424**	**−233,357**	**−220,596**	**−187,882**	**−197,296**
	RAxML-Limited	**−279,925**	**−276,859**	**−233,544**	**−220,750**	**−187,972**	**−197,373**
Quicktree	RAxML	**−275,718**	**−274,076**	**−235,199**	**−220,745**	**−185,701**	**−188,468**
	FastTree	**−276,428**	**−274,407**	**−235,246**	**−220,845**	**−185,846**	**−188,597**
	RAxML-Limited	**−276,645**	**−274,878**	**−235,385**	**−221,000**	**−185,966**	**−188,615**

ML scores given as log likelihoods; 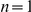 for each reported value.

Since the relative performance with respect to tree error to some extent depended upon the size of the datasets, we discuss results starting with the three largest datasets before discussing the smaller datasets. Since the reference tree for all the biological datasets is RAxML on the curated alignment, we expect RAxML to have lower tree error on the reference alignment than RAxML-Limited and FastTree.

#### The three largest datasets, 16S.B.ALL, 16S.T, and 16S.3

**Figure 3:**
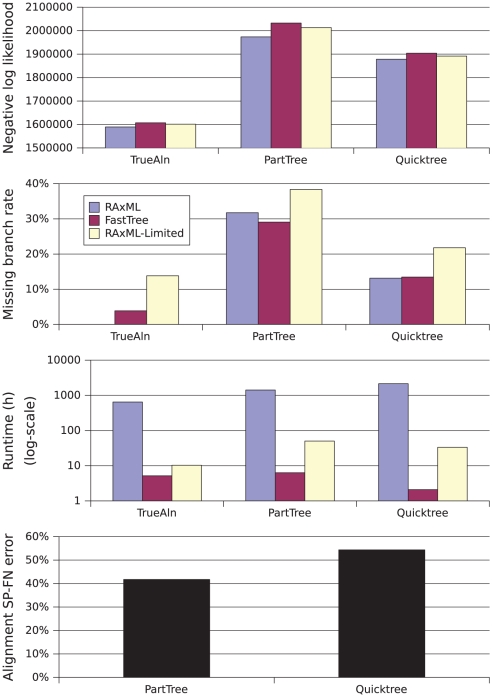
Comparison of ML methods on the 16S.B.ALL dataset. GTRGAMMA ML scores, missing branch rates, runtimes in hours, and alignment SP-FN errors are shown. 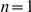 for each reported value.

On the largest dataset, 16S.B.ALL (27,643 sequences), only the Quicktree and PartTree alignment methods could be run [7]. The other alignment methods aborted due to excessive memory usage, even when provided with 256 GB of main memory. Our comparisons for this dataset are therefore based upon only three alignments. On the true alignment, RAxML had the lowest error (0.0%), FastTree had 3.9% missing branch rate, and RAxML-Limited had 13.8% (Fig. 3). The relative performance on the QuickTree alignment showed similar trends, with RAxML having 13.2% missing branch rate, followed closely by FastTree at 13.5%, and then by RAxML-Limited at 21.8%. Interestingly, on the PartTree alignment, RAxML was no longer in first place: it had 31.8% error, FastTree had 29.1%, and RAxML-Limited had 38.4%. Overall, on this dataset, RAxML-Limited had the worst accuracy by far, and RAxML and FastTree were closer (only 4% apart for each alignment, with FastTree better on one relatively inaccurate alignment). A running time comparison shows a huge difference among these methods (see Fig. 3): RAxML was the slowest, taking between 647 and 2150 hours to produce trees, FastTree the fastest, taking between 2 and 6.3 hours, and RAxML-Limited in between, taking between 10 and 50 hours.

The next two largest datasets, 16S.3 and 16S.T, have 6323 and 7350 sequences, respectively, and represent comparable challenges. For these datasets we were able to obtain alignments from all five alignment methods, the sole exception being MAFFT on the 16S.3 dataset, which failed due to memory requirements on a machine with 32 GB of main memory.

RAxML produced more accurate trees than RAxML-Limited on all alignments, with differences ranging from small (about 1.5%) to large (about 6%) (Table 3). RAxML produced more accurate trees than FastTree on all but two alignments, FastTree was better than RAxML on one alignment (the PartTree alignment of the 16S.T dataset), and they were tied on one alignment (Quicktree on 16S.T). The difference in tree error was never more than 3.2% for the curated alignment of the 16S.3 dataset. Furthermore, differences on the estimated alignments were always less than 1%. Running times differed here as well (see Table 4): RAxML took 73–305 hours, FastTree took at most 4.1 hours, and RAxML-Limited took at most 5.1 hours on each alignment.

**Table 3:** Missing branch rates (%) and alignment SP-FN errors of ML methods on alignments the three largest biological datasets.

Missing branch rate (%)					
Alignment	ML Method	16S.B.ALL	16S.T	16S.3	Average
TrueAln	RAxML	0.0	0.0	0.0	0.0
	FastTree	3.9	2.8	3.2	3.3
	RAxML-Limited	13.8	5.5	6.1	8.4
SATé	RAxML	n.d.	7.5	6.8	n.a.
	FastTree	n.d.	8.2	7.7	n.a.
	RAxML-Limited	n.d.	11.0	8.4	n.a.
MAFFT	RAxML	n.d.	7.3	n.d.	n.a.
	FastTree	n.d.	8.2	n.d.	n.a.
	RAxML-Limited	n.d.	8.9	n.d.	n.a.
PartTree	RAxML	31.8	17.1	12.0	20.3
	FastTree	29.1	16.3	12.5	19.3
	RAxML-Limited	38.4	18.6	15.4	24.1
ClustalW	RAxML	n.d.	9.7	9.9	n.a.
	FastTree	n.d.	10.5	10.4	n.a.
	RAxML-Limited	n.d.	12.9	13.3	n.a.
Quicktree	RAxML	13.2	33.9	31.8	26.3
	FastTree	13.5	33.9	32.5	26.6
	RAxML-Limited	21.8	35.0	35.6	30.8

Alignment SP-FN error (%)					
Alignment		16S.B.ALL	16S.T	16S.3	
SATé		n.d.	37.0	24.9	30.9
MAFFT		n.d.	31.0	n.d.	31.0
Quicktree		54.4	63.0	52.8	56.7
ClustalW		n.d.	56.3	52.0	54.2
PartTree		41.7	34.3	22.6	32.9

**Only three alignment methods succeeded on the three largest biological datasets. For the other alignments that could not be successfully computed due to excessive memory requirements, topological accuracy of the ML methods were not available and thus not reported.** “Average” refers to the average across the datasets. Entries for which no data was available because the alignment method failed to complete are marked “n.d.”. Averages that were not computed due to missing alignments for some datasets are marked “n.a.”. 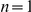 for each reported value other than the average.

**Table 4:** Runtime (h) of ML methods on alignments of the three largest biological datasets.

Alignment	ML Method	16S.B.ALL	16S.T	16S.3	Average
TrueAln	RAxML	647.3	305.3	322.1	424.9
	FastTree	5.2	1.0	1.1	2.4
	RAxML-Limited	10.3	3.7	3.1	5.7
SATé	RAxML	n.d.	123.6	123.1	n.a.
	FastTree	n.d.	1.7	0.8	n.a.
	RAxML-Limited	n.d.	2.7	1.0	n.a.
MAFFT	RAxML	n.d.	188.3	n.d.	n.a.
	FastTree	n.d.	1.3	n.d.	n.a.
	RAxML-Limited	n.d.	1.4	n.d.	n.a.
PartTree	RAxML	1418.1	176.2	118.3	570.9
	FastTree	6.3	4.1	2.4	4.3
	RAxML-Limited	50.3	5.1	2.9	19.4
ClustalW	RAxML	n.d.	73.0	64.3	n.a.
	FastTree	n.d.	0.7	0.6	n.a.
	RAxML-Limited	n.d.	1.0	0.8	n.a.
Quicktree	RAxML	2149.9	247.3	120.7	839.3
	FastTree	2.1	0.9	0.7	1.2
	RAxML-Limited	33.3	1.6	1.3	12.1

“Average” refers to the average across datasets. **Some alignments were missing due to the inability of an alignment method to successfully align a dataset due to excessive memory requirements on datasets of this size, preventing ML methods from running;** these entries are marked “n.d.”. “n.a.” denotes averages that were not computed due to missing entries. 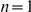 for values in columns other than the “Average” column. 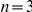 for the “Average” column.

#### Results on the seven smaller biological datasets (117–1028 sequences)

As before, RAxML produced more accurate trees than RAxML-Limited, and RAxML-Limited produced the least accurate trees (Table 5). The relative performance between FastTree and RAxML changed for these data, however. FastTree produced topologically more accurate trees on several of the alignments of these datasets, including one in which the difference in tree error was almost 10% (Quicktree on the 23S.E.aa_ag dataset). The largest differences in tree error were on the smallest datasets, where they were as large as 10%. However, the difference in tree error between FastTree and RAxML was at most 3% on the alignments of the two largest of these datasets with 901 and 1028 sequences. Running time differences were substantial here as well (Table 6). On the two largest datasets in this collection, 16S.M.aa_ag and 16S.M, each with around 1000 sequences, RAxML used between 3.4 and 10 hours, compared to at most 10 minutes for FastTree and 20 minutes for RAxML-Limited. On the other datasets, each of which has at most 300 sequences, RAxML used up to 2.7 hours, compared to at most 5 minutes for FastTree and 17 minutes for RAxML-Limited.

**Table 5:** Missing branch rates (%) and alignment SP-FN errors ML methods on alignments of the six smallest biological datasets.

Missing branch rate (%)								
Alignment	ML Method	16S.M.aa_ag	16S.M	23S.M	23S.M.aa_ag	23S.E.aa_ag	23S.E	Average
TrueAln	RAxML	0.7	0.0	0.0	0.0	0.0	0.0	0.1
	FastTree	1.6	0.7	5.4	3.2	3.3	9.3	3.9
	RAxML-Limited	3.7	3.8	11.9	5.1	12.1	10.7	7.9
SATé	RAxML	4.8	6.9	10.7	11.5	9.9	4.0	8.0
	FastTree	6.7	6.2	10.7	12.8	9.9	10.7	9.5
	RAxML-Limited	9.0	9.3	14.3	15.4	12.1	13.3	12.2
MAFFT	RAxML	4.4	5.5	11.3	10.3	8.8	8.0	8.0
	FastTree	7.4	5.7	10.7	9.6	13.2	12.0	9.8
	RAxML-Limited	8.8	6.2	16.7	13.5	13.2	20.0	13.0
PartTree	RAxML	12.7	8.8	22.0	19.9	14.3	6.7	14.1
	FastTree	12.9	8.6	22.6	18.6	17.6	12.0	15.4
	RAxML-Limited	12.9	10.0	22.6	19.9	19.8	17.3	17.1
ClustalW	RAxML	13.9	11.2	16.7	16.0	22.0	17.3	16.2
	FastTree	12.7	8.6	16.7	14.7	23.1	20.0	16.0
	RAxML-Limited	11.3	11.4	16.7	15.4	23.1	22.7	16.8
Quicktree	RAxML	10.4	10.2	20.8	19.9	28.6	24.0	19.0
	FastTree	11.5	9.3	19.6	17.9	18.7	20.0	16.2
	RAxML-Limited	10.6	10.0	20.2	17.9	25.3	20.0	17.3

Alignment SP-FN error (%)								
Alignment		16S.M.aa_ag	16S.M	23S.M	23S.M.aa_ag	23S.E.aa_ag	23S.E	Average
SATé		22.7	22.0	29.3	28.4	22.2	21.2	24.3
MAFFT		22.6	21.8	28.6	28.3	19.5	18.5	23.2
Quicktree		37.5	41.0	48.4	43.8	26.6	28.1	37.6
ClustalW		38.2	42.6	46.2	47.6	30.0	38.5	40.5
PartTree		23.1	27.5	32.1	33.8	20.7	19.7	26.1

“Average” refers to the average across the six datasets. 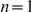 for each reported value other than the average.

**Table 6:** Runtime (h) of the ML methods on the six smallest biological datasets.

Alignment	Method	16S.M.aa_ag	16S.M	23S.M	23S.M.aa_ag	23S.E.aa_ag	23S.E	Average
Reference	RAxML	7.54	5.90	2.33	2.25	0.99	0.78	3.30
	FastTree	0.08	0.06	0.04	0.03	0.02	0.02	0.04
	RAxML-Limited	0.24	0.22	0.10	0.10	0.04	0.03	0.12
SATé	RAxML	6.26	4.51	1.57	1.37	0.67	0.62	2.50
	FastTree	0.14	0.06	0.03	0.03	0.02	0.02	0.05
	RAxML-Limited	0.21	0.18	0.07	0.07	0.03	0.03	0.10
MAFFT	RAxML	4.27	3.99	1.56	2.28	0.88	0.53	2.25
	FastTree	0.07	0.06	0.03	0.03	0.02	0.01	0.04
	RAxML-Limited	0.16	0.16	0.08	0.29	0.03	0.03	0.12
PartTree	RAxML	10.05	7.19	2.71	1.86	0.85	0.76	3.90
	FastTree	0.16	0.11	0.05	0.08	0.02	0.02	0.07
	RAxML-Limited	0.33	0.28	0.09	0.08	0.03	0.03	0.14
ClustalW	RAxML	5.99	3.73	1.64	2.10	0.66	0.51	2.44
	FastTree	0.05	0.04	0.02	0.02	0.01	0.01	0.03
	RAxML-Limited	0.14	0.11	0.06	0.06	0.02	0.02	0.07
Quicktree	RAxML	5.46	3.37	1.48	1.38	0.90	0.58	2.19
	FastTree	0.05	0.04	0.02	0.02	0.01	0.01	0.03
	RAxML-Limited	0.19	0.12	0.05	0.05	0.03	0.02	0.08

“Average” refers to the average across the datasets. *n* = 1 for values in columns other than the “Average” column. *n* = 6 for the “Average” column.

#### Summary for results

The study showed that RAxML produced better ML scores than both FastTree and RAxML-Limited, and topologically more accurate trees than RAxML-Limited, in almost all cases. However, the relative performance of FastTree and RAxML depended upon the alignment and dataset, so that RAxML typically produced slightly more accurate trees than FastTree on the large datasets. Results on smaller datasets showed larger differences in tree error, and also showed FastTree sometimes producing more accurate trees than RAxML (typically on inaccurate alignments). Running time differences were particularly pronounced on the larger datasets, but FastTree was faster than RAxML on all datasets by at least two orders of magnitude.

### Comparison to the Price *et al.* study [10]

We now compare our study to Price *et al.*'s study [10], in which RAxML was observed to consistently produce better ML scores and more accurate trees than FastTree (with error rates that differed by at least 3.6%), and to be 100–1000 times slower than FastTree.

Our results are in agreement with [10] with respect to running time and ML scores, but made somewhat different observations about tree error differences. For example, Price et al. always found RAxML to produce topologically more accurate trees than FastTree, and we found that FastTree was sometimes more accurate than RAxML. The most likely explanation for this difference is that Price et al. only used true alignments to perform this evaluation (a condition in which our study also showed RAxML to be more accurate), and we used both true and estimated alignments. Another difference between these two studies is that the difference in tree error was somewhat larger for Price et al. (where the error rate difference ranged from 3.6% to 4.7%), while we generally saw smaller differences. This difference between our studies is interesting, and raises the question of whether FastTree's simplifying heuristics are not as well suited to amino-acid phylogeny (which is what the Price et al. study examined) as nucleotide phylogeny (which is what we examined). Since our study did not examine protein-coding nucleotide sequences, it is also possible that the differences might be due to the kind of nucleotide sequences we examined (i.e., non-coding), and that RAxML and FastTree might differ substantially on coding sequences.

## Discussion

Our study examined the relative performance of two variants of RAxML and FastTree on nucleotide datasets, including several very large biological datasets (one with almost 28,000 sequences) and simulated datasets with 1000 sequences. The results of our study establish the following. First, RAxML clearly produces better ML scores compared to RAxML-Limited and FastTree, and topologically more accurate trees than RAxML-Limited, in almost all cases. When used with highly accurate alignments, RAxML also tends to produce topologically more accurate trees than FastTree, but the differences tend to be small on large datasets. When used with less accurate alignments (such as might be estimated on very large datasets, on which the most accurate alignment methods cannot run [7]), FastTree is competitive with RAxML with respect to tree topology accuracy, and can sometimes be more accurate. However, as has been observed before [10], RAxML is computationally much more expensive, taking from 100–1000 times as much time as FastTree. Thus, while RAxML produces better ML scores, and more accurate trees than FastTree in many cases, whether RAxML should be used on large datasets must be considered in the light of its increased computational requirements.

Our study is limited to nucleotide sequences, and therefore the relative performance between RAxML and FastTree could be different on amino-acid sequences. Furthermore, phylogenetic ML methods provide estimations of branch lengths and other numeric model parameters, and it is possible that the improved ML scores obtained by RAxML reflect improved estimations in these other model parameters. For applications such as detecting selection in which the other model parameters are important, improved model parameter estimation, such as might be enabled by using RAxML, may be necessary.

Future studies should investigate whether RAxML produces improved estimations of other model parameters, and the impact of these improved estimations. It would also be beneficial to evaluate whether RAxML and FastTree differ when amino-acid alignments must be estimated, since Price *et al.* only considered true alignments.

## Materials and Methods

All datasets used in this study are previously published, and are available (along with the reference tree and alignment) at http://www.cs.utexas.edu/users/phylo/datasets, in the Dryad Digital Repository, doi:10.5061/dryad.n9r3h, or in TreeBASE [15]. The simulated datasets and biological datasets, other than 16S.B.ALL, were studied in [8]. The 16S.B.ALL dataset was studied in [7], [9].

### Method commands and version numbers

Multiple sequence alignments were produced using MAFFT (using its L-INS-i and PartTree algorithms) version 6.240 [16]–[18], ClustalW (using its default and Quicktree options) version 2.0.4, and SATé version 1.1 alpha [7], [8] (available from www.cs.utexas.edu/users/phylo/datasets/tol/tol.html). We used the following commands with these programs:

- MAFFT L-ins-i default:
  mafft –localpair –maxiterate 1000
  –quiet <input>><output>
- MAFFT PartTree:
  mafft –parttree –retree 2
  –partsize 1000 <input>><output>
- ClustalW default:
  clustalw2 -align -infile = <input>
  -outfile = <output> -output = fasta
- ClustalW Quicktree:
  clustalw2 -align -infile = <input>
  -outfile = <output> -output = fasta
  -quicktree
- SATé :
  ./sate_basic.pl -r <name of run>
  -w <empty temporary work directory with full path>
  -d <input unaligned sequences file with full path>
  -l 1 -s 1 -a 5

To perform ML analyses, we used RAxML version 7.2.6 and FastTree version 2.1.3. The following commands were used to run these programs:

- RAxML (and RAxML-Limited):
  raxmlHPC -m GTRCAT -w <work dir>
  -n <identifying suffix> -s <input> -j
- FastTree:
  FastTree -nt -gtr -nosupport
  -log <log file> <input alignment>><output tree>

Where necessary, we parallelized the RAxML analyses by either recompiling with PTHREADS and using the flag -T <number of threads> or by recompiling with MPI; the parallelization did not otherwise affect the RAxML commands. RAxML's outputs were unaffected by parallelization, and all reported runtimes are for serialized execution.

### Computational Resources

For all datasets except the three largest biological datasets, SATé and two-phase analyses were performed using a heterogeneous Condor [19] computing cluster at the University of Texas at Austin. This cluster had computers with between 1 and 8 cores running at speeds between 1.86 GHz and 3.16 GHz. All programs were run as 32-bit serial executables on a single dedicated core with dedicated access to at least 512 MB and at most 4 GB of main memory.

To run SATé and the two-phase methods on the 16S.T and 16S.3 datasets, we used a 64-bit computing cluster at the University of Texas at Austin, consisting of machines with 8-core 2.83 GHz Intel Xeon 64-bit CPUs with 32 GB main memory per CPU. Due to the memory requirements of SATé and the two-phase methods on the 16S.B.ALL dataset, we used two machines with very large shared memory, each having a 16-core 64-bit AMD Opteron CPU running at 2.5 GHz, and with either 128 GB or 256 GB main memory.

## Supporting Information

Figure S1 **ML scores of ML methods on alignments of simulated 1000-taxon datasets.** ML scores are computed using RAxML, and then normalized by the ML score obtained on the RAxML(MAFFT) topology. Standard error bars are shown. *n* = 20 for each reported value. Using one-tailed pairwise t-tests with Benjamini-Hochberg correction [13], RAxML's ML score is a statistically significant improvement over FastTree's ML score for all alignments of all model conditions except SAT'e on 1000L5 (*α* = 0.05 and *n* = 40 for each test). Using similar tests, FastTree's ML score is a statistically significant improvement over RAxML-Limited's ML score for all alignments of all model conditions (*α* = 0.05 and n = 40 for each test).
(EPS)

Table S1 **Simulation parameters and empirical statistics for the 1000-taxon datasets.** The model conditions varied the gap length distribution, the probability of a gap event, and the model tree height according to the simulation procedure described in [8] and [9]. Definitions for the empirical statistics are given in Table S7.
(TIF)

Table S2
**Q-values from statistical tests comparing the missing branch rates of FastTree and RAxML-Limited.** One-tailed pairwise t-tests were used to check if FastTree's missing branch rate was a significant improvement over RAxML-Limited's missing branch rate. 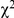 tests were used to test if the proportion of datasets showing improvement in FastTree's missing branch rate over RAxML-Limited's missing branch rate differed from the proportion of datasets not showing such an improvement. All q-values were corrected for multiple comparisons using the Benjamini-Hochberg method [13]. *n* = 40 for each test.(TIF)

Table S3 **Q-values from one-tailed pairwise t-tests comparing the missing branch rates of RAxML and FastTree.** One-tailed pairwise t-tests were used to check if RAxML's missing branch rate was a significant improvement over FastTree's missing branch rate. Similar tests were used to check if FastTree's missing branch rate was a significant improvement over RAxML's missing branch rate. All q-values were corrected for multiple comparisons using the Benjamini-Hochberg method [13]. *n* = 40 for each test.
(TIF)

Table S4
**Q-values from statistical tests comparing the ML scores of RAxML and FastTree.** One-tailed pairwise t-tests were used to check if RAxML's ML score was a significant improvement over FastTree's ML score. 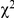 tests were used to test if the proportion of datasets showing improvement in RAxML's ML score over FastTree's ML score differed from the proportion of datasets not showing such an improvement. All q-values were corrected for multiple comparisons using the Benjamini-Hochberg method [13]. *n* = 40 for each test.(TIF)

Table S5
**Q-values from statistical tests comparing the ML scores of FastTree and RAxML-Limited.** One-tailed pairwise t-tests were used to check if FastTree's ML score was a significant improvement over RAxML-Limited's ML score. 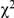 tests were used to test if the proportion of datasets showing improvement in FastTree's ML score over RAxML-Limited's ML score differed from the proportion of datasets not showing such an improvement. All q-values were corrected for multiple comparisons using the Benjamini-Hochberg method [13]. *n* = 40 for each test.(TIF)

Table S6
**Q-values from** 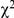 **tests comparing the missing branch rates of RAxML and FastTree.** 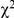 tests were used to test if the proportion of datasets showing improvement in RAxML's missing branch rate over FastTree's missing branch rate differed from the proportion of datasets not showing such an improvement. All q-values were corrected for multiple comparisons using the Benjamini-Hochberg method [13]. *n* = 40 for each test.(TIF)

Table S7 **Empirical statistics for the biological datasets.** From left to right, the dataset name, number of taxa, number of aligned sites in the reference alignment, resolution of the reference tree, average and maximum p-distance of the reference alignment, percentage of the reference alignment consisting of indels, and average gap length of the reference alignment are shown. The resolution of the reference tree is the number of internal edges in the reference tree divided by the maximum possible number of internal edges in the reference tree (which is *n* (3, for n the number of taxa). The p-distance between two aligned sequences is defined as the percentage of sites for which the two sequences have differing nucleotides. The average p-distance in an alignment is the average p-distance for all pairs of aligned sequences in the alignment, and the maximum p-distance in an alignment is the maximum p-distance for any pair of aligned sequences in the alignment.
(TIF)
