# Supplementary figures and images for: RAxML and FastTree: Comparing Two Methods for Large-Scale Maximum Likelihood Phylogeny Estimation
Source: PLoS One. 2011 Nov 21;6(11):e27731. doi: 10.1371/journal.pone.0027731 (PMC3221724; doi:10.1371/journal.pone.0027731)

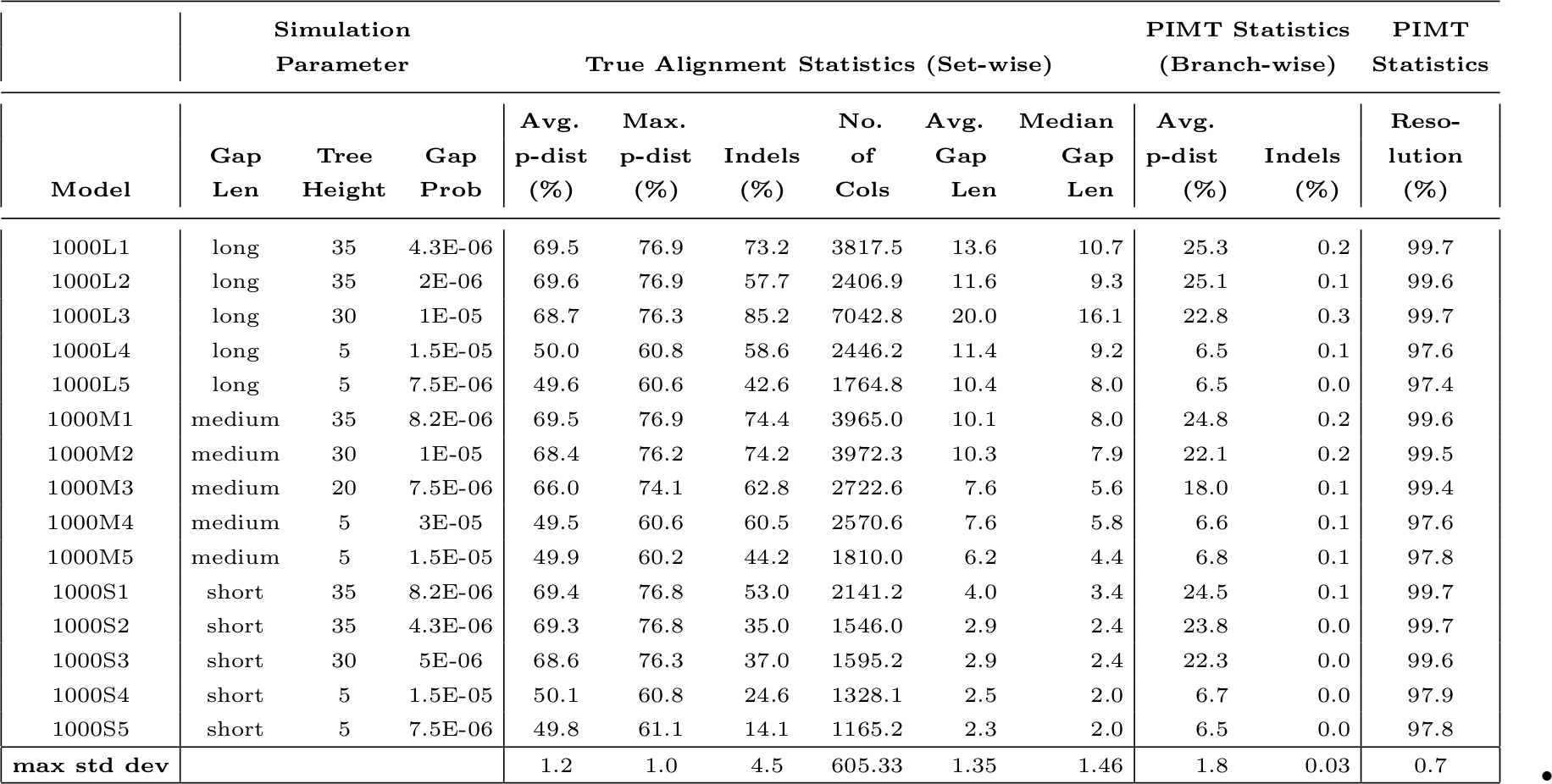

Supplement: Table S1 — Simulation parameters and empirical statistics for the 1000-taxon datasets. The model conditions varied the gap length distribution, the probability of a gap event, and the model tree height according to the simulation procedure described in [8] and [9]. Definitions for the empirical statistics are given in Table S7. (TIF) [file pone.0027731.s002.tif]

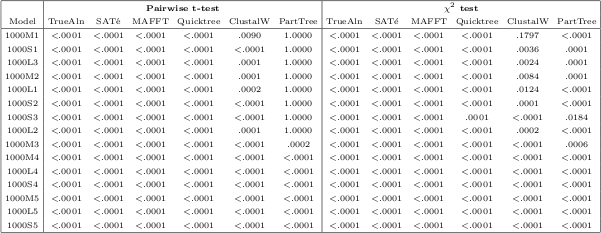

Supplement: Table S2 — Q-values from statistical tests comparing the missing branch rates of FastTree and RAxML-Limited. One-tailed pairwise t-tests were used to check if FastTree's missing branch rate was a significant improvement over RAxML-Limited's missing branch rate. tests were used to test if the proportion of datasets showing improvement in FastTree's missing branch rate over RAxML-Limited's missing branch rate differed from the proportion of datasets not showing such an improvement. All q-values were corrected for multiple comparisons using the Benjamini-Hochberg method [13]. n = 40 for each test. (TIF) [file pone.0027731.s003.tif]

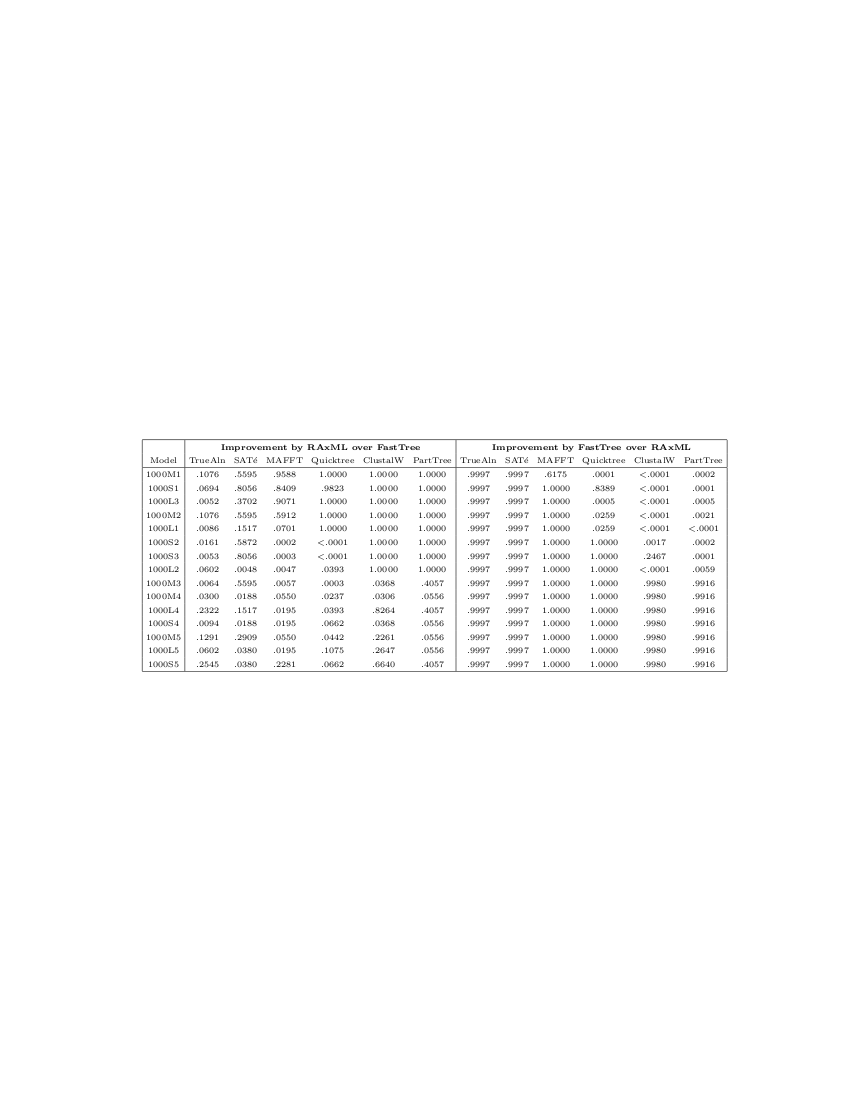

Supplement: Table S3 — Q-values from one-tailed pairwise t-tests comparing the missing branch rates of RAxML and FastTree. One-tailed pairwise t-tests were used to check if RAxML's missing branch rate was a significant improvement over FastTree's missing branch rate. Similar tests were used to check if FastTree's missing branch rate was a significant improvement over RAxML's missing branch rate. All q-values were corrected for multiple comparisons using the Benjamini-Hochberg method [13]. n = 40 for each test. (TIF) [file pone.0027731.s004.tif]

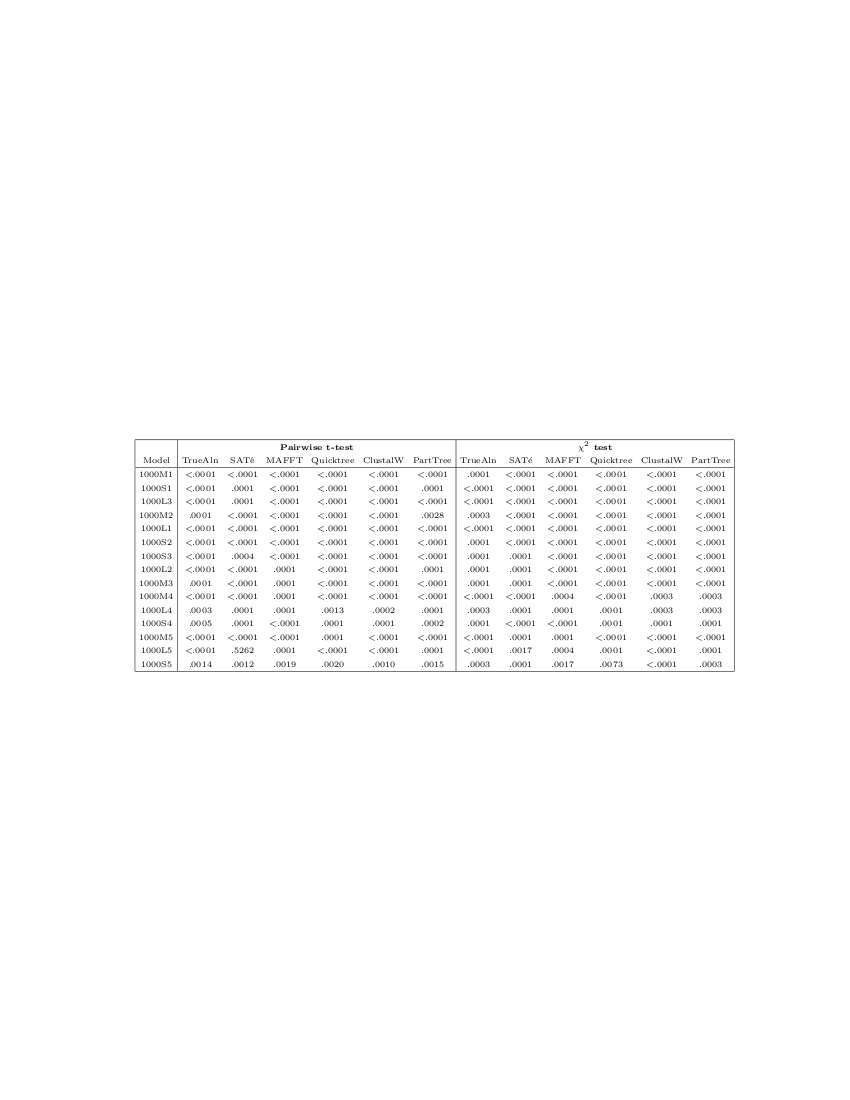

Supplement: Table S4 — Q-values from statistical tests comparing the ML scores of RAxML and FastTree. One-tailed pairwise t-tests were used to check if RAxML's ML score was a significant improvement over FastTree's ML score. tests were used to test if the proportion of datasets showing improvement in RAxML's ML score over FastTree's ML score differed from the proportion of datasets not showing such an improvement. All q-values were corrected for multiple comparisons using the Benjamini-Hochberg method [13]. n = 40 for each test. (TIF) [file pone.0027731.s005.tif]

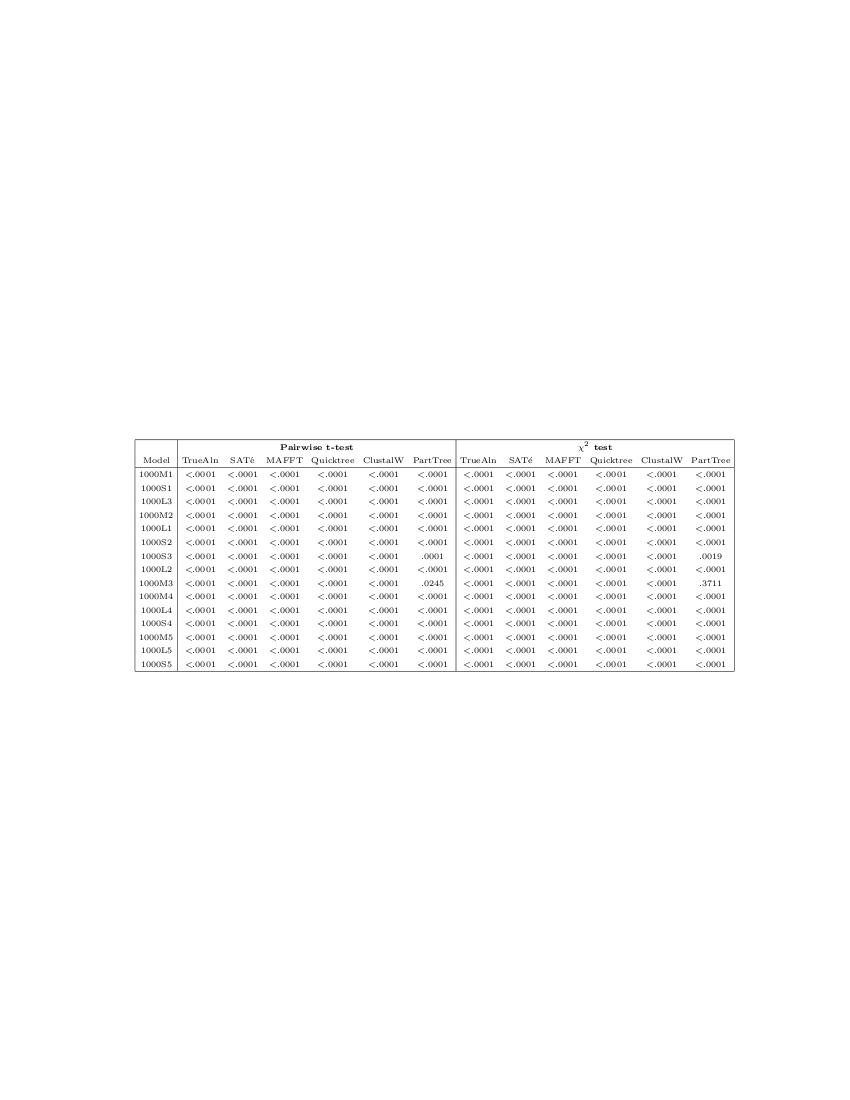

Supplement: Table S5 — Q-values from statistical tests comparing the ML scores of FastTree and RAxML-Limited. One-tailed pairwise t-tests were used to check if FastTree's ML score was a significant improvement over RAxML-Limited's ML score. tests were used to test if the proportion of datasets showing improvement in FastTree's ML score over RAxML-Limited's ML score differed from the proportion of datasets not showing such an improvement. All q-values were corrected for multiple comparisons using the Benjamini-Hochberg method [13]. n = 40 for each test. (TIF) [file pone.0027731.s006.tif]

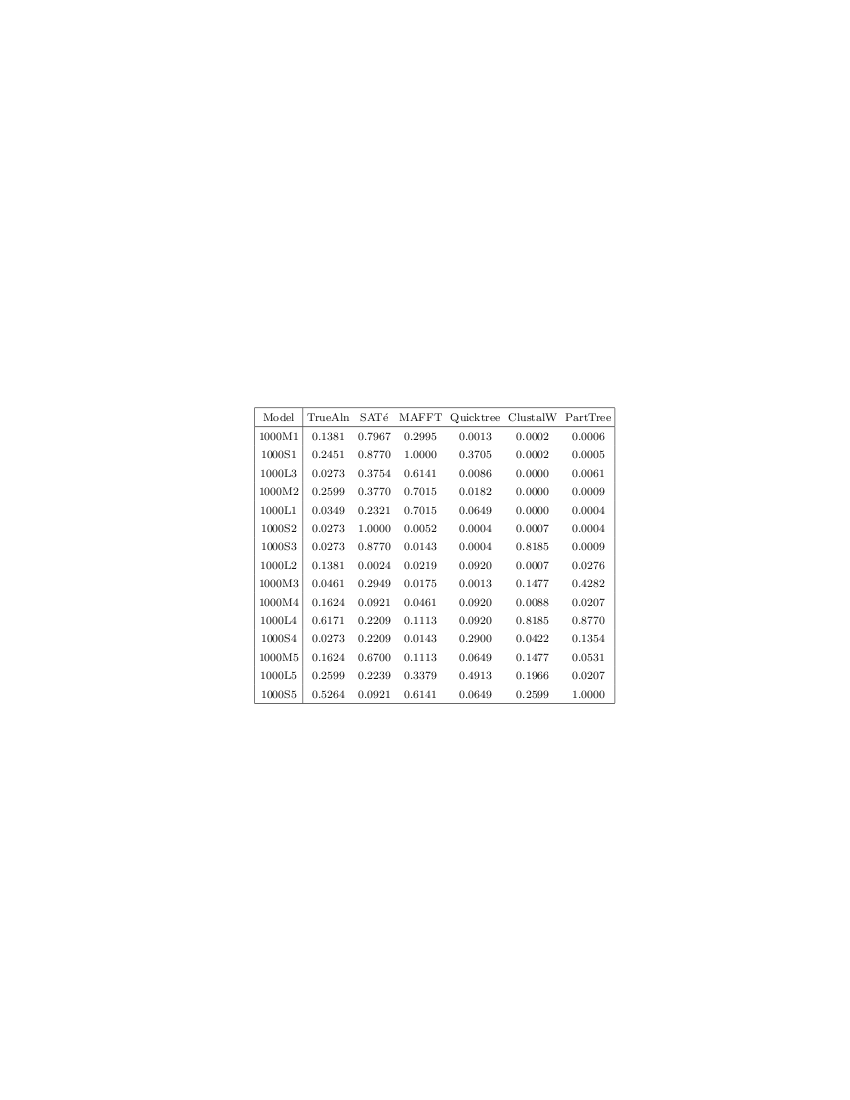

Supplement: Table S6 — Q-values from tests comparing the missing branch rates of RAxML and FastTree. tests were used to test if the proportion of datasets showing improvement in RAxML's missing branch rate over FastTree's missing branch rate differed from the proportion of datasets not showing such an improvement. All q-values were corrected for multiple comparisons using the Benjamini-Hochberg method [13]. n = 40 for each test. (TIF) [file pone.0027731.s007.tif]

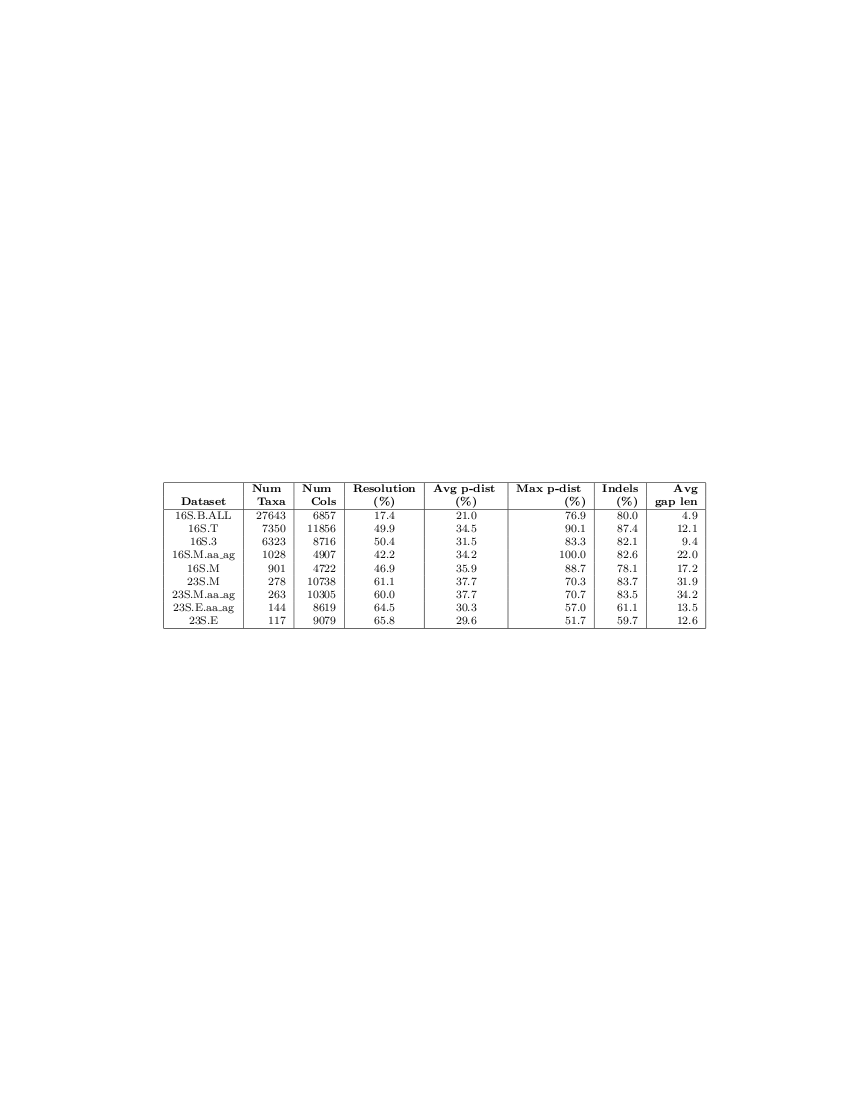

Supplement: Table S7 — Empirical statistics for the biological datasets. From left to right, the dataset name, number of taxa, number of aligned sites in the reference alignment, resolution of the reference tree, average and maximum p-distance of the reference alignment, percentage of the reference alignment consisting of indels, and average gap length of the reference alignment are shown. The resolution of the reference tree is the number of internal edges in the reference tree divided by the maximum possible number of internal edges in the reference tree (which is n (3, for n the number of taxa). The p-distance between two aligned sequences is defined as the percentage of sites for which the two sequences have differing nucleotides. The average p-distance in an alignment is the average p-distance for all pairs of aligned sequences in the alignment, and the maximum p-distance in an alignment is the maximum p-distance for any pair of aligned sequences in the alignment. (TIF) [file pone.0027731.s008.tif]
